# The effect of polymer stiffness on magnetization reversal of magnetorheological elastomers

**DOI:** 10.1063/5.0086761

**Published:** 2022-04-13

**Authors:** Andy T. Clark, David Marchfield, Zheng Cao, Tong Dang, Nan Tang, Dustin Gilbert, Elise A. Corbin, Kristen S. Buchanan, Xuemei M. Cheng

**Affiliations:** 1 Department of Physics, Bryn Mawr College, Bryn Mawr, Pennsylvania 19010, USA; 2 Department of Physics, Colorado State University, Fort Collins, Colorado 80523, USA; 3 Department of Biomedical Engineering, University of Delaware, Newark, Delaware 19716, USA; 4 Materials Science and Engineering, University of Tennessee, Knoxville, Tennessee 37996, USA; 5 Department of Material Science and Engineering, University of Delaware, Newark, Delaware 19716, USA; 6 Nemours/Alfred I. duPont Hospital for Children, Wilmington, Delaware 19803, USA

## Abstract

Ultrasoft magnetorheological elastomers (MREs) offer convenient real-time magnetic field control of mechanical properties that provides a means to mimic mechanical cues and regulators of cells *in vitro*. Here, we systematically investigate the effect of polymer stiffness on magnetization reversal of MREs using a combination of magnetometry measurements and computational modeling. Poly-dimethylsiloxane-based MREs with Young’s moduli that range over two orders of magnitude were synthesized using commercial polymers Sylgard^™^ 527, Sylgard 184, and carbonyl iron powder. The magnetic hysteresis loops of the softer MREs exhibit a characteristic pinched loop shape with almost zero remanence and loop widening at intermediate fields that monotonically decreases with increasing polymer stiffness. A simple two-dipole model that incorporates magneto-mechanical coupling not only confirms that micrometer-scale particle motion along the applied magnetic field direction plays a defining role in the magnetic hysteresis of ultrasoft MREs but also reproduces the observed loop shapes and widening trends for MREs with varying polymer stiffnesses.

## I. INTRODUCTION

Magnetorheological elastomers (MREs) are multifunctional materials that consist of a non-magnetic elastomeric matrix with embedded micro- or nano-sized magnetic particles. The elastic moduli^1–8^ and surface roughness^8–12^ of MREs can be tuned using an applied magnetic field, where mechanical changes of several orders of magnitude have been reported. In addition, the base elastic moduli at zero magnetic field of MREs can span across several orders of magnitude, depending on the constituent polymer types as well as the type and concentration of the embedded magnetic particles.^13^ MREs have consequently become attractive for a wide range of applications in the automotive industry, construction, electronics, biology, medicine, and robotics.^14^

Recently, ultrasoft MREs with a base Young’s modulus (*E*) of several kPa have received great attention because they offer an innovative means to mimic biophysical mechanical cues and regulators of cells *in vitro*.^6–8^ Ultrasoft MREs have shown much larger magnetic field-dependent changes in their moduli^6,8^ than what was predicted by the analytic models that consider stationary magnetic dipoles.^15,16^ Unlike rubber-like MREs, soft MREs have been shown to exhibit magnetic field-dependent motion of the constituent magnetic particles within the polymer matrix.^17,18^ The magnetic hysteresis loops of soft MREs are also markedly different than those of stiffer MREs and exhibit a characteristic pinched loop shape with zero remanence and loop widening at intermediate fields.^19^ Particle motion is thought to be an important contributing factor to this loop shape,^20–23^ and recent experiments on hysteresis loops in an MRE that is stiffened by lowering the temperature provide compelling evidence that the magnetic particle motion is, indeed, linked to the widening of the magnetic hysteresis loops.^24–26^ However, the temperature-dependent experiments to date^24–26^ only examine two stiffnesses and a more comprehensive examination of the effect of stiffness that includes experiments and modeling is needed.

In this work, we investigate the effect of polymer stiffness on magnetization reversal of MREs where the elastic moduli are varied systematically over the range from ultrasoft to rubber-like by varying the polymer composition. While cooling an ultrasoft polymer^24–26^ has the advantage that the measurements can be performed on the same sample, only two stiffnesses can be reliably accessed. Our measurements cover a wide range of MRE stiffnesses, and we further confirm that hysteresis loops measured in the same ultrasoft MRE at low temperatures where the polymer is rubber-like are identical to the room temperature hysteresis loops from rubber-like MREs synthesized with stiffer polymers. We also compare our measured hysteresis loops to theoretical hysteresis loops calculated using a simple two-dipole model that captures the magneto-mechanical coupling in MREs. Our modeling approach is similar to approaches used recently in the field,^20–23^ using a simple description of the magnetic and elastic behavior. The modeling results reproduce the main features of the experimentally observed trends in the hysteresis measurements and provide insight into the physical mechanism of the MRE hysteresis. Our results provide evidence that the motion of magnetic particles, particularly along the direction of the applied field, plays a critical role in the magnetic hysteresis loop widening.

## II. EXPERIMENTAL DETAILS

Ultrasoft (*E* ≈ kPa) poly-dimethylsiloxane (PDMS)-based MREs were synthesized by mixing Sylgard^™^ 527 (Dow Corning^™^) polymer with carbonyl iron powder (BASF^™^) at volume fractions of *Φ* = 3%, 23%, 30%, and 40%. To investigate the effect of stiffness on magnetic properties, harder MREs with *E* that range over two orders of magnitude were synthesized^27^ by adding different amounts of a harder Sylgard^™^ 184 polymer, as shown in Table I. We note that unless otherwise indicated, *E* refers to Young’s modulus at zero magnetic field. Samples for magnetometry measurements were cut from the center of the as-prepared MREs to a size of 4 × 4 × 1 mm^3^. See the supplementary material for more details. Major magnetic hysteresis loops of MRE samples at room temperature were measured using a Lakeshore Cyrotronics^™^ Micromag 3900 vibrating sample magnetometer (VSM) by decreasing the magnetic field *H* applied in the sample plane from 15 to −15 kOe and then increasing back to 15 kOe with a field sweep rate of 100 Oe/s, where 15 kOe is well above the saturation field for all the MRE samples. Temperature-dependent major magnetic hysteresis loops with *H* cycled between ±15 kOe and minor hysteresis loops with *H* cycled between ±5 kOe with a field sweep rate of 20 Oe/s for MRE sample 1 were measured at selective temperatures between 300 and 2 K by a Quantum Design^™^ PPMS VSM. In particular, the sample was field-cooled (FC) at 5 kOe for the minor loops measured at lower temperatures. The field sweep rates were chosen to provide sufficient time for iron particles within the MREs to respond to the magnetic field change (see the supplementary material).

## III. MAGNETIZATION REVERSAL OF MREs

**FIG. 1.**
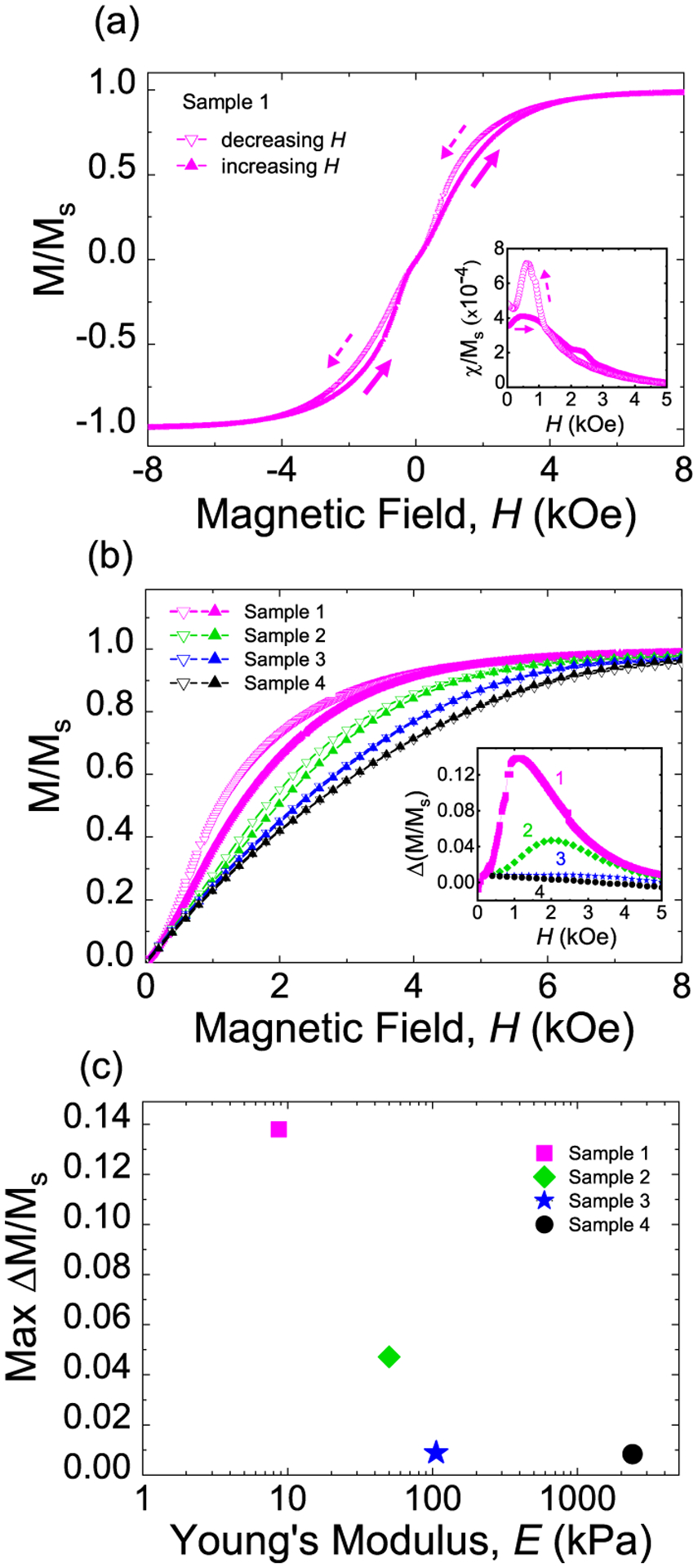
Room temperature magnetic reversal of MREs. (a) The major hysteresis loop of ultrasoft MRE sample 1 shows zero remanent magnetization and a characteristic loop widening at the intermediate fields. The inset compares the normalized differential susceptibility *χ*/*M*_*s*_ for the decreasing and increasing *H* branches, where a five-point average was applied to reduce random noise. (b) The zoomed-in view of the first quadrant of the major hysteresis loops for MRE samples 1–4 having polymer stiffnesses ranging from ultrasoft (1) to rubber-like (4). The inset shows the field dependence of Δ(*M*/*M*_*s*_), the difference between the magnetizations for the increasing and decreasing branches at each *H*. (c) Maximum Δ(*M*/*M*_*s*_) as a function of Young’s modulus for MRE samples 1–4.

A characteristic pinched major hysteresis loop for an ultrasoft MRE (*E* ≈9 kPa) sample is shown in Fig. 1(a) and a zoomed-in view of the first quadrant is shown as the pink curve in Fig. 1(b). While the remanence, i.e., the magnetization at zero field, is almost 0 [*M*_*r*_/*M*_*s*_ = (3.92 ± 0.01) × 10^−3^] and the coercive field is also small (*H*_*C*_ = 14 ± 1 Oe), the loop opens up at intermediate fields and closes again near the saturation field, which is referred to as loop widening. We quantify the loop widening using Δ(*M*/*M*_*s*_), which is defined as the magnetization difference of the two branches of the hysteresis loop at each *H*, as shown in the inset of Fig. 1(b). The loop widening can also be highlighted by comparing the normalized differential susceptibility *χ*/*M*_*s*_ for the decreasing *H* and increasing *H* branches, where the differential magnetic susceptibility *χ* is defined as *χ* = *dM*/*dH*, as shown in the inset of Fig. 1(a). The observed characteristic loop widening is consistent with previous reports where the authors attributed the loop widening to the magnetic particle motion in the MREs.^19,24–26^

If the observed loop widening, indeed, arises from magnetic field-dependent motion of magnetic particles within the polymer matrix, the widening should decrease with the increase in *E* of MREs, since the larger *E* will impede particle motion. To investigate the effect of polymer stiffness on magnetization reversal of MREs, we measured the major hysteresis loops for MREs with *E* ranging from ≈9 kPa (ultrasoft) to 2400 kPa (rubber-like). Figure 1(b) shows the zoomed-in view of the first quadrant of major hysteresis loops for MRE samples 1–4 with *E* as listed in Table I. The measured loop widening, characterized by Δ(*M*/*M*_*s*_), indeed, monotonically decreases with increasing *E*. The peak value of Δ(*M*/*M*_*s*_) for MRE sample 4 (stiffest) is about 10% of the peak value for MRE sample 1 (softest) as shown in Fig. 1(c).

**FIG. 2.**
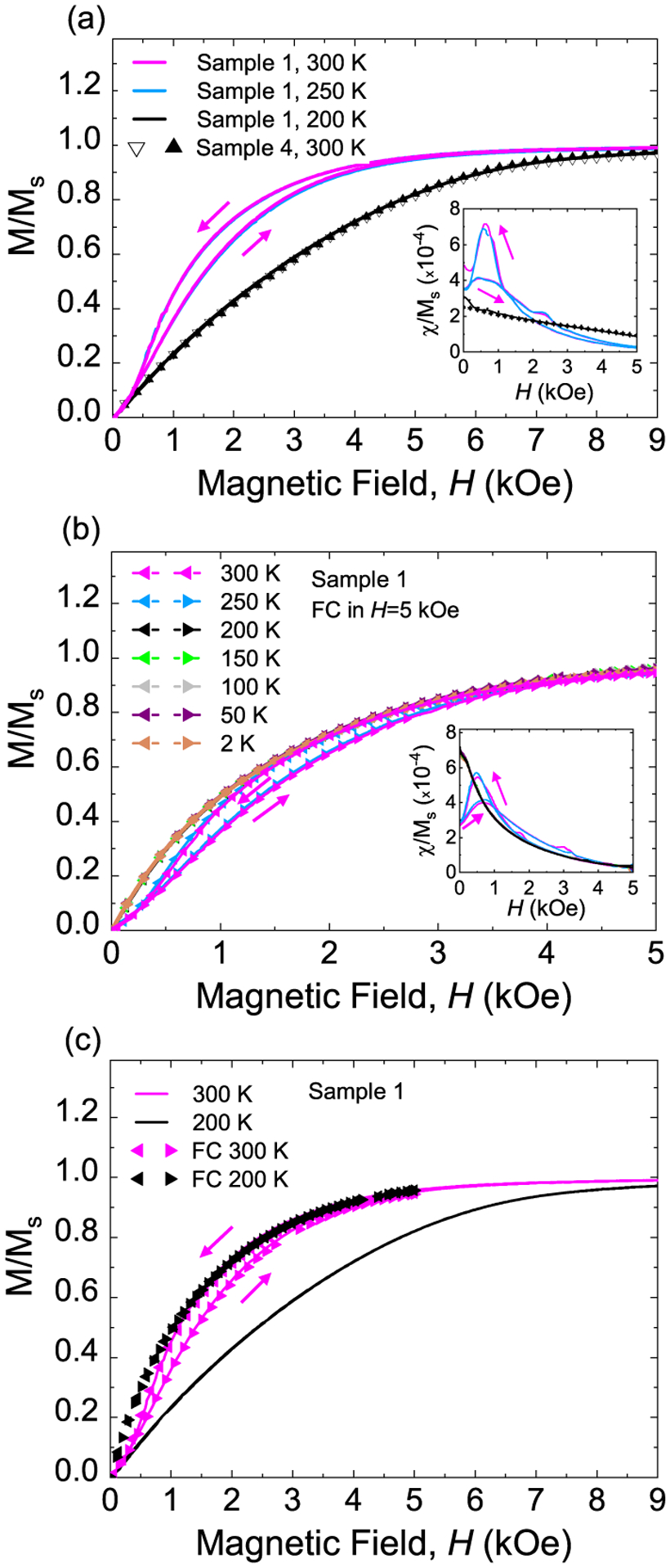
Temperature-dependent magnetic properties of MRE sample 1. (a) Zoomed-in view of the first quadrant of major hysteresis loops of MRE sample 1 measured at 300, 250, and 200 K as well as that of MRE sample 4 measured at 300 K. The inset shows the field dependence of *χ*/*M*_*s*_. (b) Field-cooled minor hysteresis loop measurements of the same ultrasoft MRE sample 1. The inset shows *χ*/*M*_*s*_ at different temperatures. (c) Comparison of major loops and FC minor loops of MRE sample 1 at temperatures above (softer) and below (stiffer) *T*_*P*_.

Temperature also provides a means to control the stiffness of an MRE since the PDMS-based MREs undergo a phase transition at *T*_*P*_ ≈ 230 K where the *E* increases by several orders of magnitude,^24–26,28^ which enables us to investigate the effects of polymer stiffness and iron particle motion on magnetization reversal in the same MRE sample. Figure 2(a) shows that while the major hysteresis loops of MRE sample 1 with ultrasoft polymer A measured at 300 and 250 K (both above *T*_*P*_) overlap and both show loop widening, the major loop of this MRE sample 1 at 200 K (below *T*_*P*_, stiffer) has no characteristic loop widening and overlaps with rubber-like MRE sample 4 (polymer D, 300 K). Figure 2(b) shows the FC-minor hysteresis loops with *H* cycled between ±5 kOe for MRE sample 1 at selected temperatures between 300 and 2 K. Similarly, all the minor loops measured above *T*_*P*_ (softer) overlap and exhibit loop widening and those measured below *T*_*P*_ (stiffer) also overlap but show no loop widening, consistent with the effect of MRE stiffness on magnetization reversal shown in Fig. 1(b).

Figure 2(c) compares the major loops and FC-minor loops of the same MRE sample 1 measured at 300 K (softer) and 200 K (stiffer). While the major and minor loops overlap at 300 K as expected, the normalized magnetization of the major loop at 200 K is significantly smaller than that of the FC-minor loops at the same field. As we explain below, this difference suggests that the magnetic particle spacing in MREs affects the magnetization reversal. Lowering the temperature increases the MRE stiffness so the particles are less movable at lower temperatures, and lowering the temperature from above to below *T*_*P*_ in *H* = 5 kOe freezes the particles at their locations from the previous FC-minor loop measured above *T*_*P*_. The magnetic particles are consequently closer together on average, resulting in stronger dipolar interactions between neighboring particles, as compared to the zero-field cooling case at 200 K for the same *H*. The difference in the normalized magnetization between the major and FC-minor loops measured below *T*_*P*_ can be further highlighted by comparing the *χ*/*M*_*s*_ values near zero field. As shown in the insets of Fig. 2, the *χ*/*M*_*s*_ near remanence for sample 1 below *T*_*P*_ is about 2.6 times larger for the minor loop as compared to the major loop, and the minor loop *χ*/*M*_*s*_ is larger than the corresponding value measured above *T*_*P*_.

**FIG. 3.**
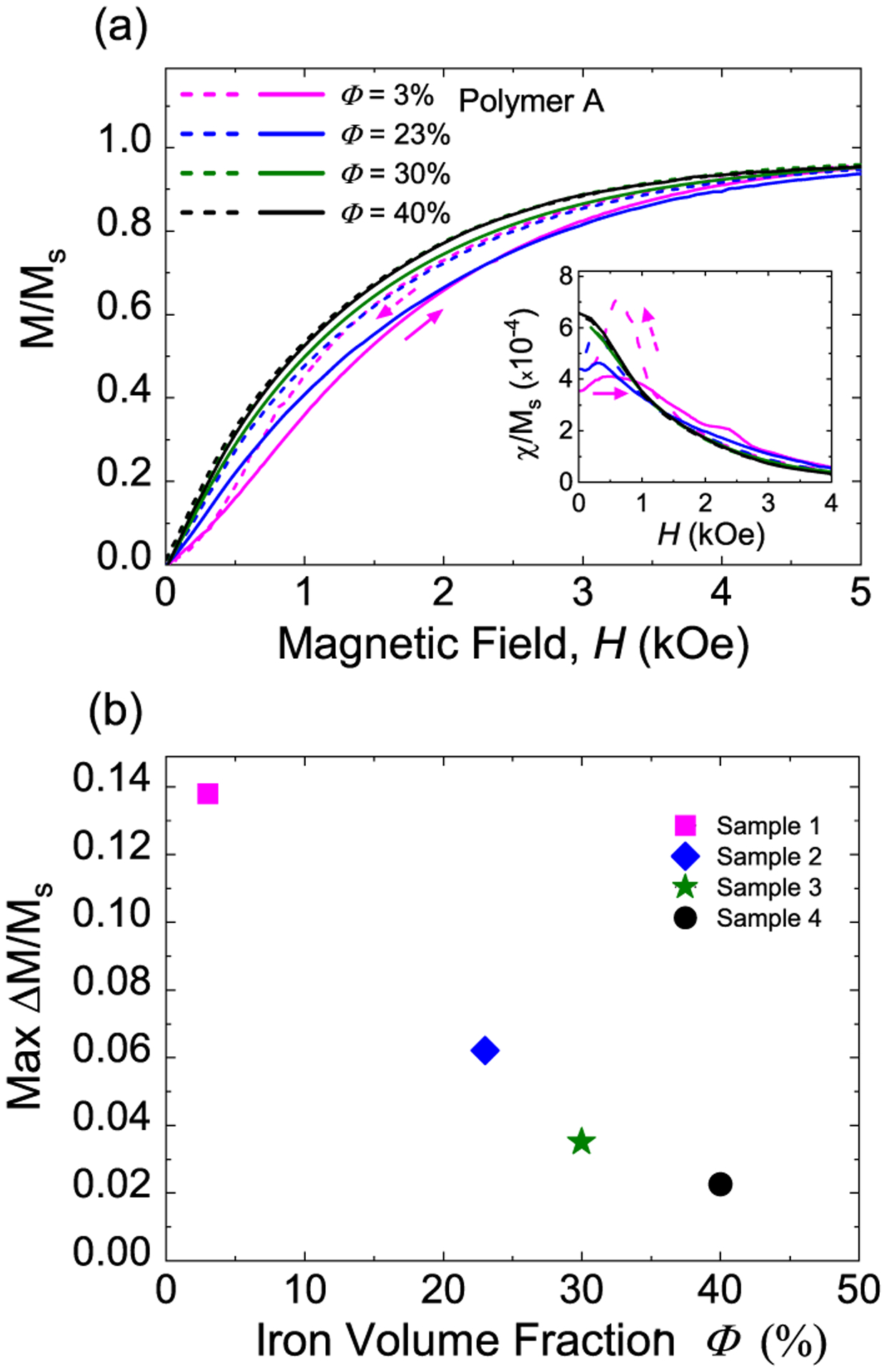
Effect of iron particle concentration on magnetic reversal of ultrasoft MREs. (a) Zoomed-in view of magnetic hysteresis loops for ultrasoft MREs containing *Φ* = 3%–40% iron particles embedded in polymer A. The inset shows the field dependence of *χ*/*M*_*s*_ where a five-point averaging was applied to reduce random noise. (b) Maximum Δ(*M*/*M*_*s*_) as a function of iron volume fraction.

Another way to modify the inter-particle spacing in MREs is to change the iron particle concentration *Φ*. To confirm the effect of magnetic particle spacing, we measured room temperature major hysteresis loops of MREs with the same polymer (A) and *Φ* ranging from 3% to 40%, as shown in Fig. 3(a). As *Φ* increases, the minimum and average inter-particle spacing both decrease so the particles have less available space to move, which results in a reduction in the loop widening [Fig. 3(b)]. Additionally, the closer distances between the iron particles lead to larger stray magnetic fields and larger magnetic moments for each particle at a given *H*. As expected, *χ*/*M*_*s*_ at remanence is higher for MREs with larger *Φ*, as shown in the inset of Fig. 3(a).

## IV. TWO-DIPOLE MAGNETO-MECHANICAL MODELING

**FIG. 4.**
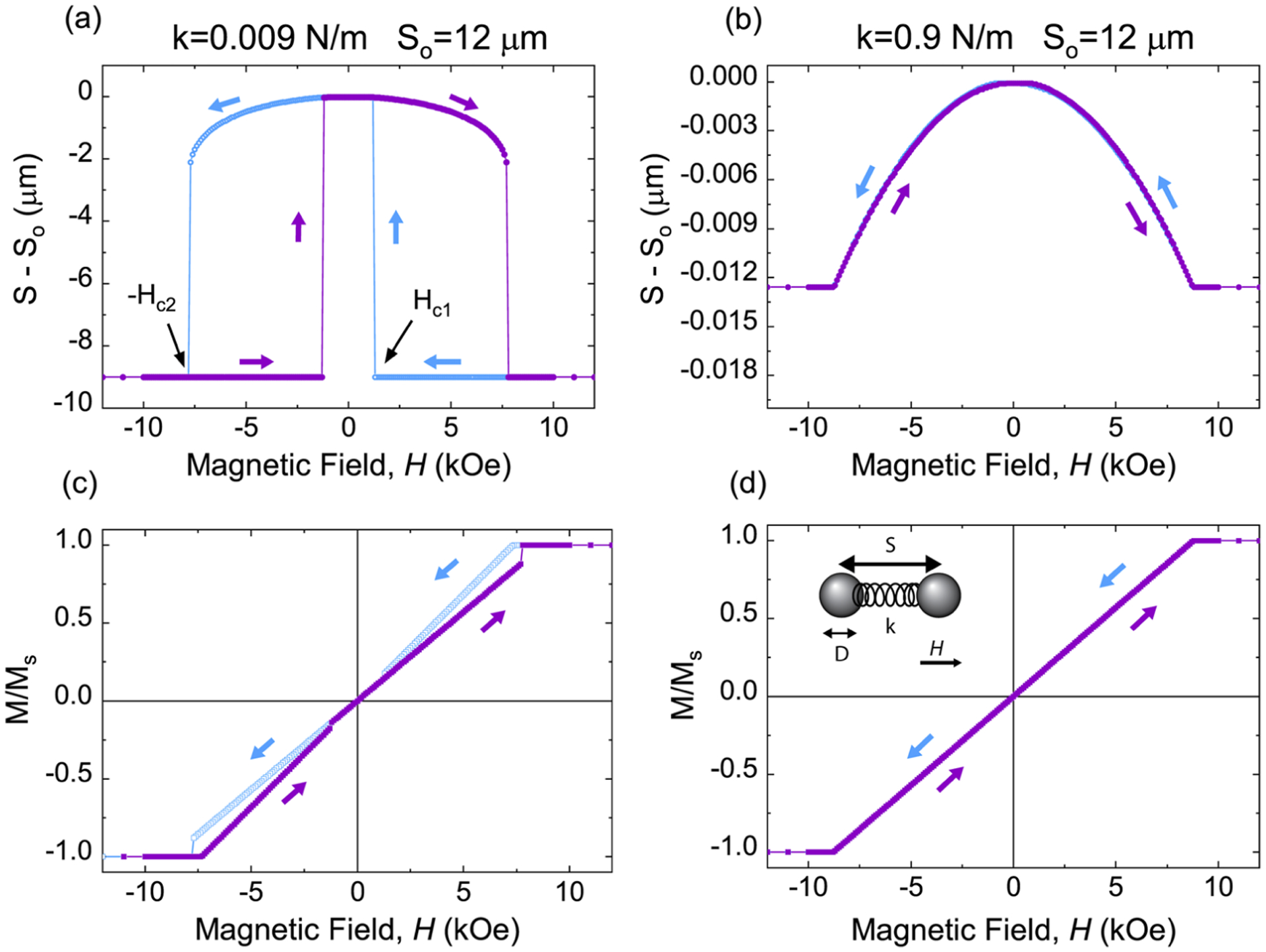
Two-dipole modeling results for two stiffness constants: *k* = 9 × 10^−3^ N/m [(a) and (c)] and *k* = 9 × 10^−1^ N/m [(b) and (d)]. In both cases, the elastic equilibrium particle separation (at zero magnetic field) is *S*_*o*_ = 12*μ*m. The inter-particle displacement (*S*_ − _*S*_*o*_) and the corresponding magnetic hysteresis loops are shown in (a) and (b) and (c) and (d), respectively. The inset of (d) shows a schematic diagram of the two-dipole model.

To further understand the effect of stiffness and particle spacing on the magnetic behavior of MREs, we used a simple two-dipole model, similar to the ball and spring modeling approach by Stepanov *et al*.^19^ and Puljiz *et al*.,^20^ to model the MRE behaviors. As illustrated in the inset of Fig. 4(d), two spherical particles of diameter *D* and saturation magnetization *M*_*s*_ are connected to each other by a single spring with a stiffness constant *k*, representing the elastic polymer. The net magnetic dipole moment of each sphere is *m* = *MV* = *χ*_*sph*_*H*_*eff*_ *V* below magnetic saturation and *m* = *M*_*s*_*V* at and above saturation, where *χ*_*sph*_ is the magnetic susceptibility of a single sphere, *H*_*eff*_ is the local effective field at the center of each particle that includes the applied field *H* and the stray field of the other sphere, and *V* = *πD*^3^/6 is the particle volume. The particles are treated as point magnetic dipoles located at the center of each sphere, and the net force experienced by either one of the spheres for the case where **H** is applied parallel to the spring is

(1) $$F=-k(S-S_{o})-\frac{3\mu_{o}m^{2}}{2\pi S^{4}},$$

where *S* is the inter-particle separation and *S*_*o*_ is the elastic equilibrium separation (also *S* = *S*_0_ at *H* = 0). A negative (positive) *F* represents an attractive (repulsive) net force. The first term in Eq. (1) is the elastic restoring force, and the second is the dipole–dipole interaction force, which is attractive when **H** is along the line connecting the two spheres. Hysteresis loops were obtained by finding the equilibrium (*F* = 0) for each *H* value where *H* was decreased from +*H*_max_ to −*H*_max_ then increased back to +*H*_max_. In practice, a nonlinear conjugate gradient method was used to find *S* associated with the local energy minimum, where the force and energy (*U*) are related by *F* = −∇*U*, and *m* is calculated at each step based on *χ*_*sph*_ and the local *H*_*eff*_. Modeling was also conducted with **H** perpendicular to the spring, which leads to a repulsive magnetic force and consequently *S* > *S*_*o*_. Modeling was conducted for selected *k* values for *S*_*o*_ = 3.2–13.0 *μ*m in steps of 0.2 *μ*m with particle diameter *D* = 3 *μ*m, *M*_*s*_ = 1.4 × 10^6^A/m, and *χ*_*sph*_ = 2. To obtain more realistic estimates of the MRE hysteresis curves, averages of the magnetic response weighted by an estimated separation distribution (a Gaussian distribution with a mean and standard deviation of 4.8 and 6.5 *μ*m, respectively) were calculated.

Figure 4 compares the particle motion and the corresponding hysteresis loops calculated for two dipoles with *S*_*o*_ = 12 *μ*m connected by a spring of different stiffness constants: *k* = 9 × 10^−3^ N/m and *k* = 9 × 10^−1^N/m, as shown in Figs. 4(a) and 4(c) and Figs. 4(b) and 4(d), respectively. The approximate equivalent *E*, obtained by considering the spring as a compressed cylinder, which yields *E* = 2*kS*_*o*_/*πD*^2^, are *E* ≈ 8 and ≈800 kPa, respectively; hence, Figs. 4(a) and 4(c) and Figs. 4(b) and 4(d) approximately correspond to the softest (sample 1) and stiffest (sample 4) MREs considered in the experiments, respectively. At large *H* where the particles are magnetically saturated, they are at their closest distance due to the attractive dipole–dipole forces. When the particles are touching, as in Fig. 4(a), we refer to this as the clustered state. As *H* is reduced, *m* decreases since *m* is proportional to *H*_*eff*_ and, consequently, the magnitude of the dipole–dipole force decreases. For the ultrasoft case in Fig. 4(a), the elastic force is small and the particles touch (*S* = *D*) at saturation. The particles remain in contact until *H* is reduced to a critical value *H*_*c*1_, where the attractive magnetic force is sufficiently small that the elastic force can pull the particles apart, as the clustered state is no longer a local minimum energy state, resulting in a jump in *S*. As *H* is further decreased to zero, *S* increases gradually to a maximum *S*_*o*_ at *H* = 0. As *H* is further decreased below zero, **H** increases in magnitude, but now in the opposite direction, the particles are attracted to each other and *S* decreases gradually at first until the particles touch once again at *H*_*c*2_ when the separated state is no longer an available minimum energy state. The corresponding magnetic response [Fig. 4(c)] shows zero remanent magnetization within the uncertainty of the calculations and exhibits a pinched loop shape that is qualitatively similar to what is observed in the experiments (Fig. 1) and also to recent modeling results for a similar system.^21^ The particle motion is reversible when *H* is removed, which is expected based on recent experiments.^20^ The field range associated with the hysteretic magnetic response (*H*_*c*1_ < |*H*| < *H*_*c*2_) corresponds to the region of bistability of particle spacings where one of the stable states corresponds to the particles touching. For larger *k* [Figs. 4(b) and 4(d)], the stronger elastic force inhibits particle contact, and there is no hysteresis in the particle motion or the magnetic response. When **H** is applied perpendicular to **S** instead of parallel to **S**, the dipole–dipole interactions are repulsive and no hysteresis is observed.

**FIG. 5.**
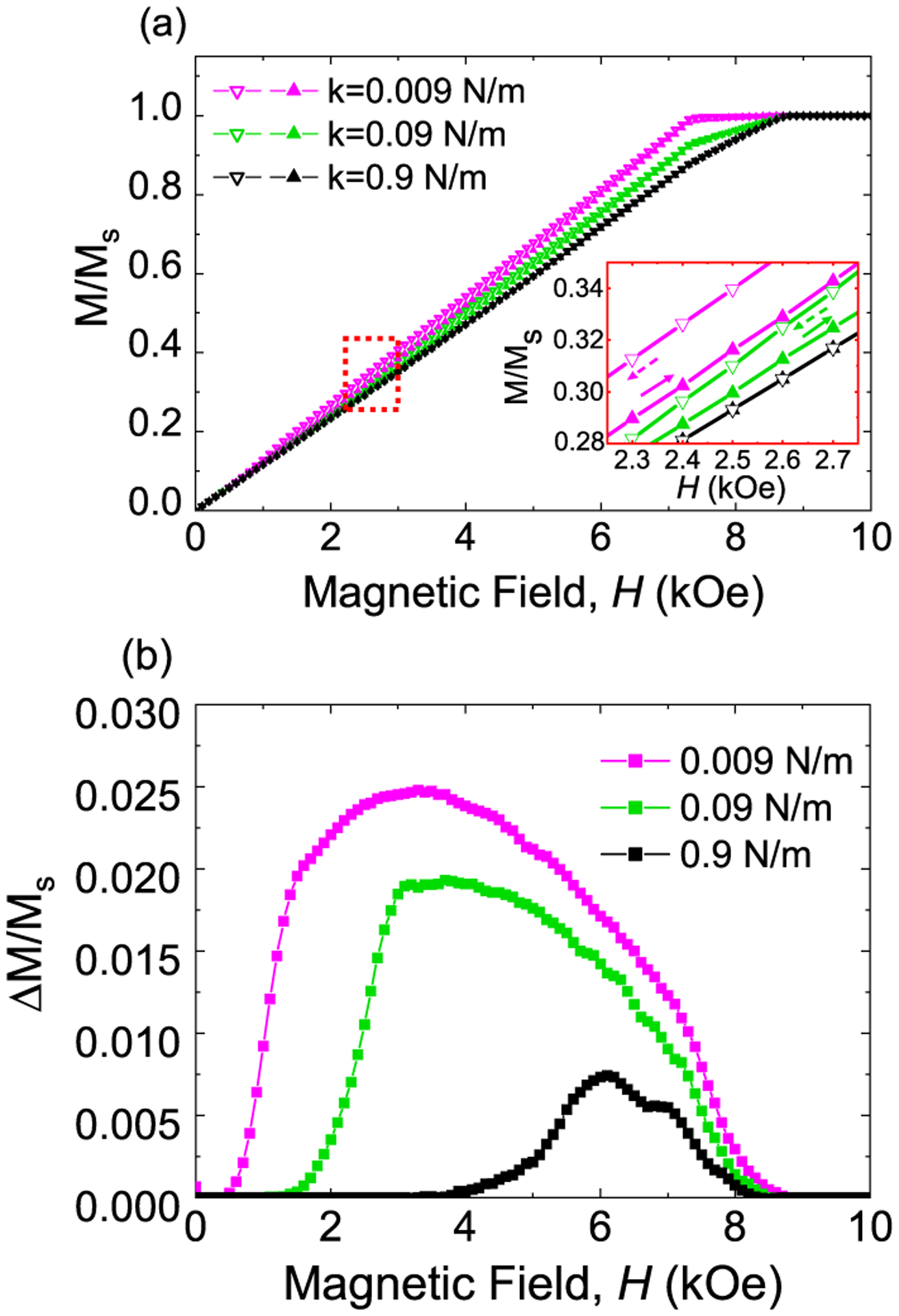
The effect of stiffness constants (*k* = 9 × 10^−1^, 9 × 10^−2^, and 9 × 10^−3^ N/m) on magnetic hysteresis loops calculated from the two-dipole model by taking weighted average of a collection of hysteresis loops calculated using a distribution of *S*_*o*_ values ranging from 3.2 to 13.0*μ*m. (a) The first quadrant of the calculated weighted average hysteresis loops; the inset shows a zoomed-in view. (b) Calculated Δ(*M*/*M*_*s*_) vs *H* for different *k*’s, where a five-point averaging was applied.

The two-dipole modeling results highlight the role of attractive inter-particle interactions in the hysteretic magnetic response. To better account for the effects of the collective behavior of an ensemble of particles, we consider a distribution of equilibrium positions, which leads to a smoother magnetic response that is more representative of a real sample. Figure 5(a) shows the zoomed-in view of the first quadrant for the weighted average of hysteresis loops calculated for *k* = 9 × 10^−1^, 9 × 10^−2^, and 9 × 10^−3^ N/m using a weighted average of hysteresis loops with *S*_*o*_ = 3.2–13*μ*m. Increasing *k* leads to a smaller loop widening, also evident in Fig. 5(b), which matches the experimentally observed trend in Fig. 1(b). Modeling also shows that increasing *k* and decreasing *S*_0_ lead to an increase in the zero-field susceptibility. Since a higher *k* and lower *S*_0_ are the expected results of the “locking in” of particles at close positions under FC conditions, this is consistent with the increase in *χ*/*M*_*s*_ at *H* = 0 observed in Fig. 2(b) as compared to Fig. 2(a) for MRE sample 1 below *T*_*P*_. A linear magnetic response is used for each sphere, which may lead to a larger *H*_*c*1_ as compared to the nonlinear response used by Biller *et al*.,^22^ and this may in part account for the lower Δ*M*/*M*_*s*_ values observed in the model as compared to the experiment.

The model of two dipoles connected by a single spring, which was used to obtain the results shown in Figs. 4 and 5, has limitations. Similar to many other models,^20–23^ the hysteretic losses from individual particles are not included although these losses should be small^19,24,29^ (see the supplementary material). Additionally, this single-spring model does not allow consideration of fields at an angle, which would cause rotation in addition to attraction/repulsion. To assess the role of fields applied at intermediate angles, we carried out additional modeling runs using a threespring approach similar to what was reported by Puljiz *et al*.^20^ with *S*_0_ = 9 *μ*m and *k* = 4 × 10^−3^ N/m for all three springs with **H** at an angle of 19^○^ (see the supplementary material). The particle response, although combined with rotation, still shows a pinched loop shape with bistability similar to what is observed in Figs. 4(a) and 4(b), and clustering is still the mechanism that leads to hysteresis. More complicated models that include additional field angles, allow particle rotation, add more particle sizes, and allow clusters of more than two particles could be important for capturing a more realistic picture of the particle motion in the MREs and for refining the shape of the hysteresis loops. However, our simple model highlights the fundamental role of the competition between the elastic and magnetic forces and the resultant local particle motion, especially the motion along the applied field direction, in the magnetization reversal of MREs. Furthermore, confocal microscopy imaging confirms that the iron particle motion in the polymer matrix is, indeed, primarily along the direction of applied magnetic field (see the supplementary material).

## V. CONCLUSION

In conclusion, we have investigated the effect of the polymer stiffness and magnetic particle spacing on the magnetization reversal of MREs experimentally and with modeling. MREs with Young’s moduli that range over two orders of magnitude were synthesized using mixtures of two polymers, Sylgard 527 and Sylgard 184, and carbonyl iron powder. Magnetometry measurements for MREs of systematically varied stiffness from ultrasoft to rubber-like show a characteristic pinched loop shape that is consistent with previous measurements on ultrasoft MREs. Our results reveal that the loop widening monotonically decreases with the increase in MRE stiffness. Furthermore, we confirm that hysteresis loops measured in the same ultrasoft MRE at low temperatures (*T* < *T*_*P*_) where the polymer is rubber-like are identical to the room temperature hysteresis loops from rubber-like MREs synthesized with stiffer polymers and the same magnetic particle volume fraction *Φ*. A two-dipole model shows that the observed loop widening arises from a bistability of inter-particle displacements along the applied magnetic field direction. This model, while simple, produces calculated magnetic hysteresis loops that show a widening trend that qualitatively matches the experimental results for MREs with varying polymer stiffnesses. Our results provide guidance for magnetic field control of MREs with a wide range of stiffnesses in biomedical and other applications.

## Supplementary Material

supplemental material

## Figures and Tables

**TABLE I.** Young’s moduli *E* of MREs with volume fraction *Φ* = 3% of iron particles synthesized using different ratios by weight of commercial polymers Sylgard^™^ 527 and Sylgard^™^ 184. Young’s moduli were measured by compressive indentation at zero magnetic field (see the supplementary material).

MRE sample	Polymer type	Sylgard^™^ 527:Sylgard^™^ 184 (by w.t.)	*E* (kPa)
1	Polymer A	1 : 0	8.7 ± 0.6
2	Polymer B	10 : 1	50 ± 2
3	Polymer C	5 : 1	106 ± 1
4	Polymer D	0 : 1	2400 ± 400

## Data Availability

The data that support the findings of this study are available from the corresponding author upon reasonable request.
