# Supplementary material for: The effect of polymer stiffness on magnetization reversal of magnetorheological elastomers
Source: APL Mater. Author manuscript; Available in PMC 2023 Feb 28. (PMC9974180; doi:10.1063/5.0086761)
Supplement: supplemental material [file NIHMS1831787-supplement-supplemental_material.docx]

**Supplementary Material: The effect of polymer stiffness on magnetization reversal of magnetorheological elastomers**

Andy T. Clark,^1^ David Marchfield,^2^ Zheng Cao,^3^ Tong Dang,^1^ Nan Tang,^4^ Dustin Gilbert,^4^ Elise A. Corbin,^3,5,6^ Kristen S. Buchanan,^2^ and Xuemei M. Cheng^1,*^

1. Department of Physics, Bryn Mawr College, Bryn Mawr, PA, USA

2. Department of Physics, Colorado State University, Fort Collins, CO, USA

3. Department of Biomedical Engineering, University of Delaware, Newark, DE, USA

4. Materials Science and Engineering, University of Tennessee, Knoxville, TN, USA

5. Department of Material Science and Engineering, University of Delaware, Newark, DE, USA

6. Nemours/Alfred I. duPont Hospital for Children, Wilmington, DE, USA

1. **MRE synthesis and magnetometry sample preparation**

Ultrasoft ($E\sim\mathrm{kPa}$) PDMS-based MREs were fabricated using Sylgard™ 527 (Dow Corning™), prepared by mixing equal parts by weight of monomer with crosslinker and then mixing in magnetically soft carbonyl iron powder (BASF™) at volume fractions of $\Phi=3,$23, 30 and 40%. After mixing, the MREs were poured into 35-mm diameter culture dishes to a thickness of ~5 mm and cured in a vacuum oven at 65^o^C for four hours.

Smaller samples were sectioned from the middle of the MREs and cut to a size of 4x4x1 mm^3^. To prevent changes in shape anisotropy due to magnetic field-dependent deformation of the MRE sample, the samples were volume-constrained by placing each sample on a silicon wafer and encasing it with a 2-part epoxy (Gorilla™).

1. **Young’s modulus measurements by compressive indentation**

The experimental Young’s modulus values listed in **Table I** were obtained by compressive indentations performed using a custom built micro-indenter similar to that described by Rennie et al^1^ and Schulze et al^2^. A spherical indenter $1.8 \mathrm{mm}$ in diameter connected to a cantilever with a spring constant of $k=1,200 N/m$ was brought into contact with the MRE surface and then immediately retracted. The Young’s modulus was calculated by fitting the unloading portion of the indentation vs. force curves with the JKR adhesive contact model.^3^ A plot of *E* as a function of Sylgard 184 percentage is shown in **Fig. S1**. The measurements were all done with no magnetic field applied.


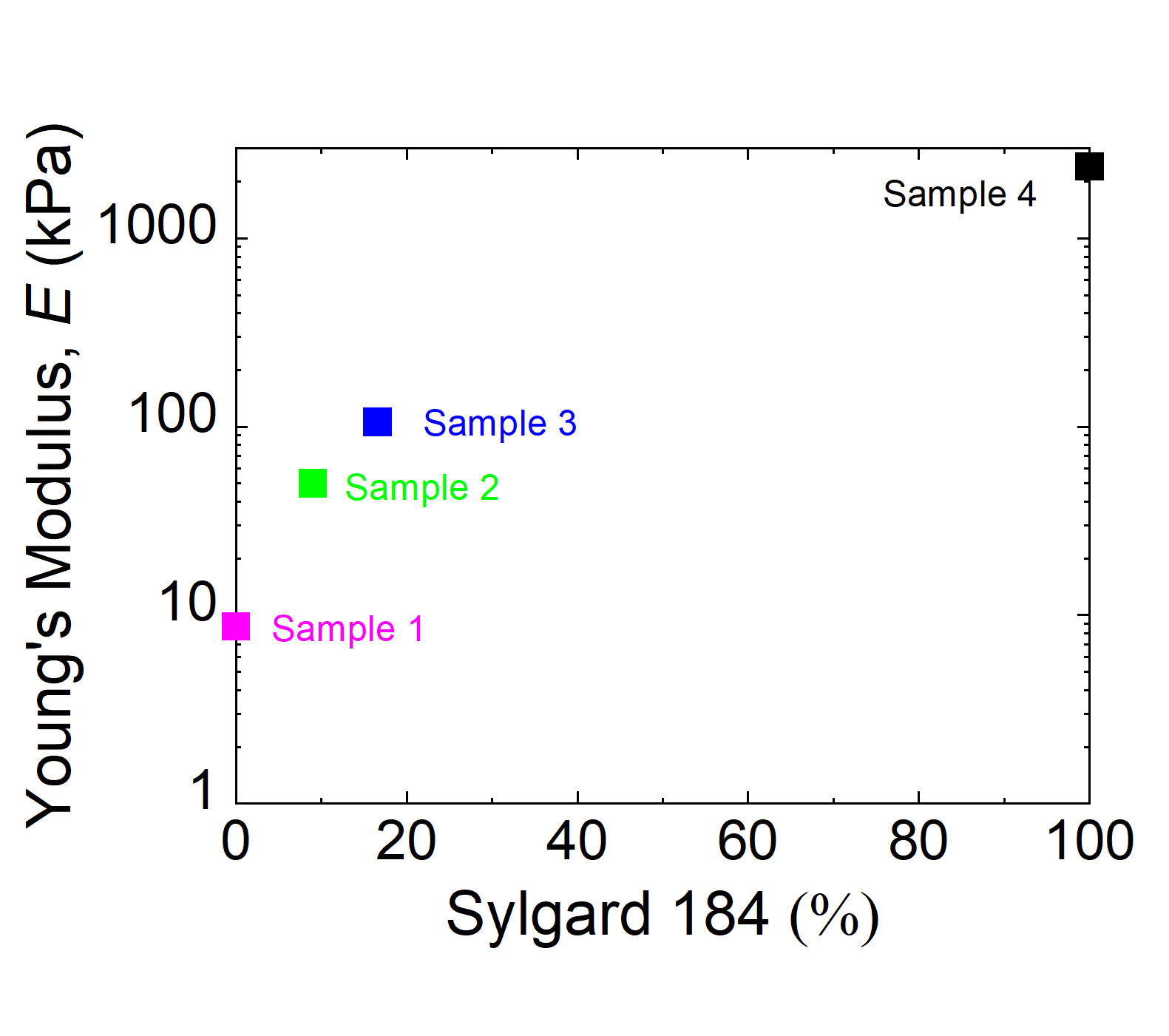


**Fig. S1** Young’s modulus *E* of MRE samples 1-4 synthesized using commercial polymer Sylgard™ 527 with different amounts of added harder Sylgard™ 184 polymer by weight measured at $H=0$.

1. **Additional results of magnetometry measurements**

A. M-H Loop of Carbonyl Iron Powder

The magnetic hysteresis of carbonyl iron powder was measured by magnetometry. **Fig. S2a** shows the major magnetic hysteresis loop for carbonyl iron powder with a zoomed-in view of the first quadrant in **Fig. S2b**. The hysteresis loop shows no loop widening, confirming that the individual iron particles have negligible hysteresis loss.


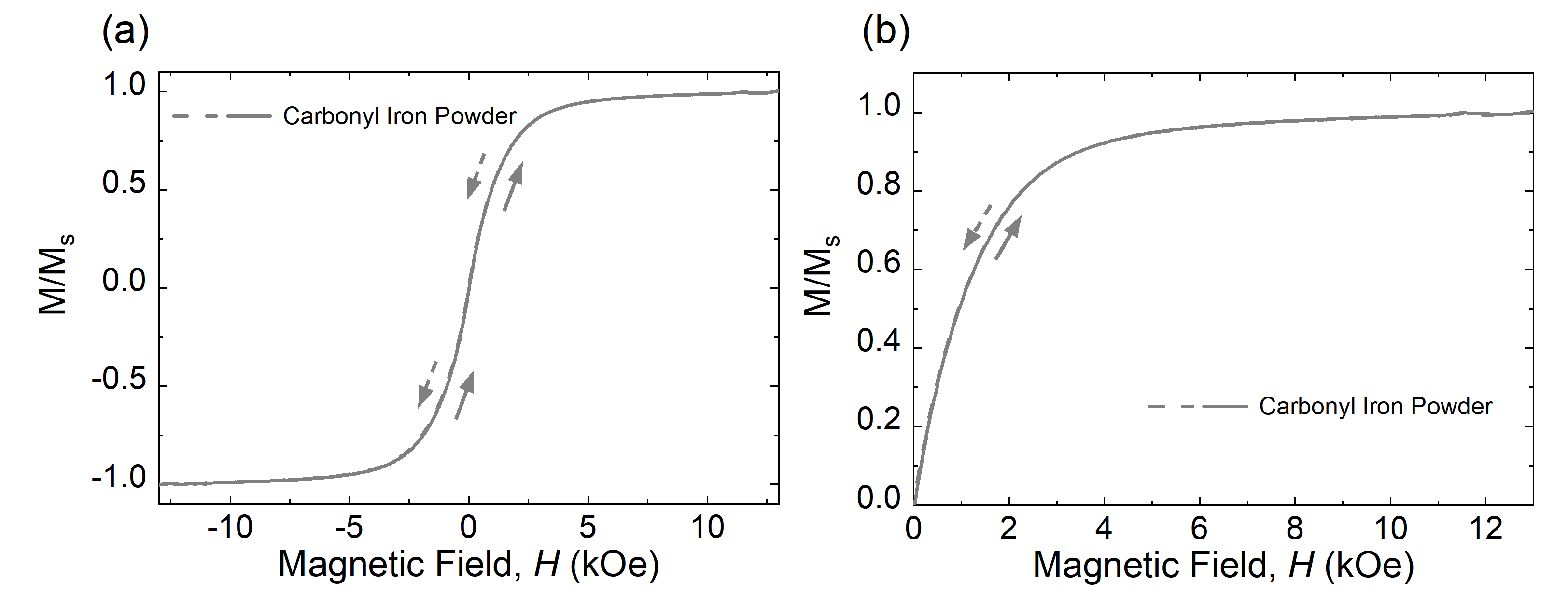


**Fig. S2** Magnetic hysteresis of carbonyl iron powder. (a) Major magnetic hysteresis loop of carbonyl iron powder. (b) Zoomed-in view of the first quadrant of the major magnetic hysteresis loop of carbonyl iron powder.

B. M-H Loops of unconstrained vs. constrained MREs

Ultrasoft MREs exhibit magnetic field-dependent changes in sample shape upon application of an external magnetic field. To determine whether macroscopic sample deformation and the resultant shape anisotropy changes affect the hysteresis loop of the ultrasoft MREs considered in this study, major magnetic hysteresis loop measurements were made on the ultrasoft MRE sample 1 before and after the sample shape was constrained. **Fig. S3** shows a zoomed-in view of the first quadrant of the major magnetic hysteresis loops which are identical, hence constraining the shape of the MRE and thereby restricting changes in shape anisotropy, has no effect on the observed loop widening.


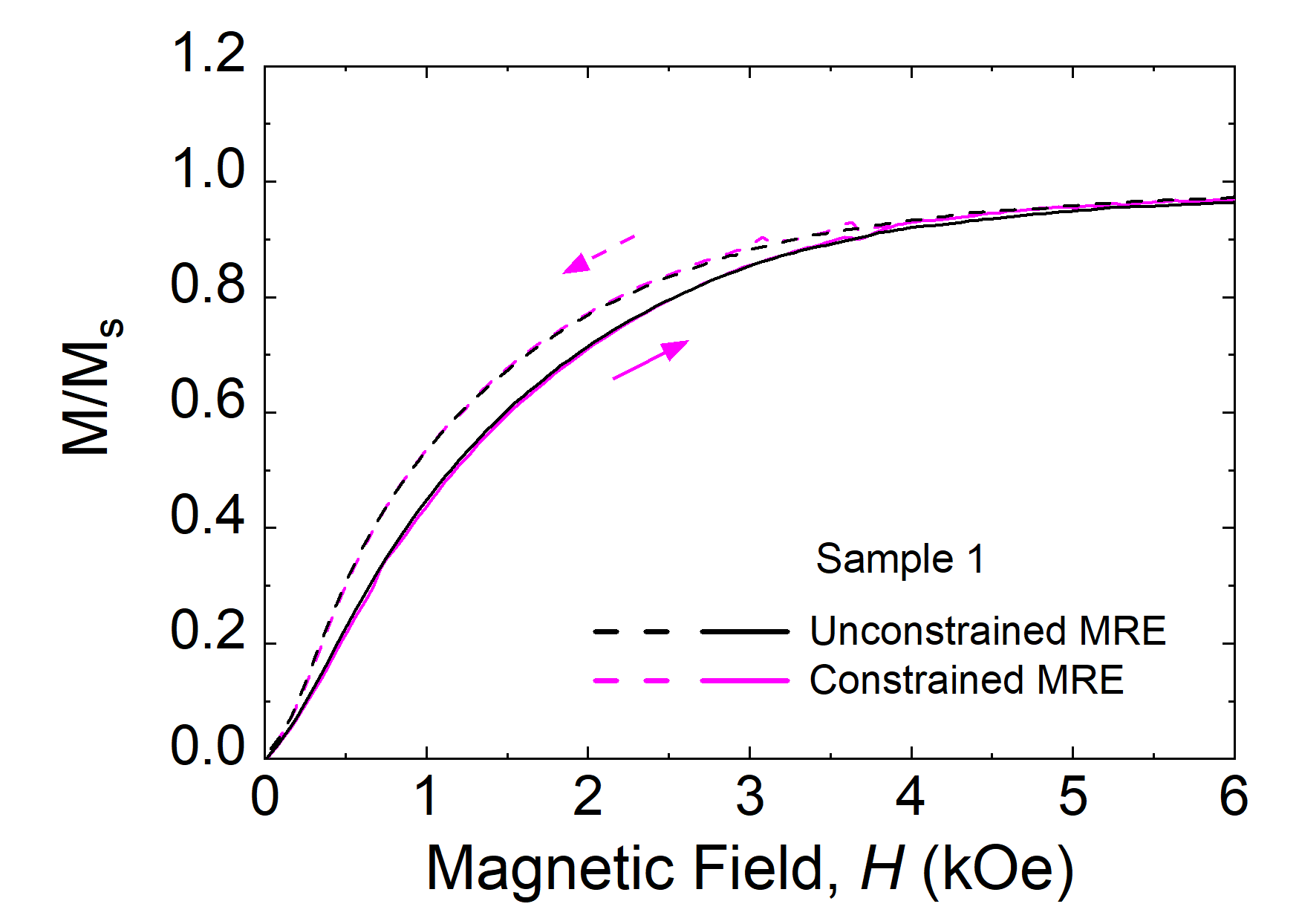


**Fig. S3** Zoomed-in view of the first quadrant of the major magnetic hysteresis loops for the MRE sample 1 before and after the sample shape was constrained.

C. Effect of Magnetic Field Sweep Rate on M-H Loops of MREs

The effect of field sweep rate on the observed widening in the hysteresis loops of ultrasoft MREs was investigated by magnetometry. Major magnetic hysteresis loops for MRE sample 1 were measured for varying magnetic field sweep rates ranging from 500 – 20 Oe/s as shown in **Fig. S4a**. A zoomed-in view of the first quadrant displayed in **Fig. S4b** where the inset shows the field-dependence of $\Delta(M/M_{s})$. The hysteresis loops show a 35% decrease in the peak $\Delta(M/M_{s})$ as the sweep rate is decreased from 500 Oe/s down to 100 Oe/s, after which minimal difference is observed.


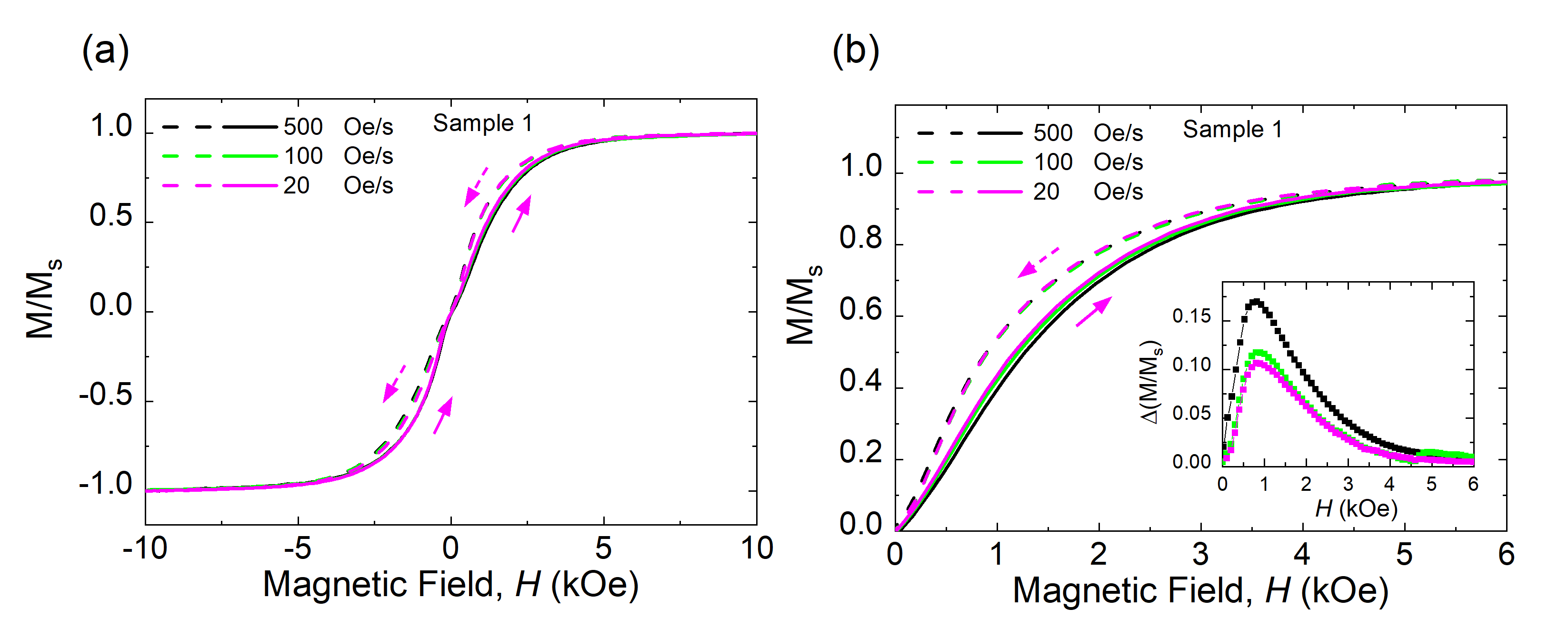


**Fig. S4** Comparison of the major magnetic hysteresis loops for the MRE sample 1 measured with varying magnetic field sweep rates. (a) Major magnetic hysteresis loop for varying magnetic field sweep rate. (b) Zoomed-in view of the first quadrant of the magnetic hysteresis loops with inset showing the field-dependence of the calculated $\Delta(M/M_{s})$ for each sweep rate.

1. **3D Particle Tracking by Magnetic Field-Dependent Confocal Microscopy**

The magnetometry and modeling results in the main paper indicate that local particle motion is important in the magnetization reversal process in ultrasoft MREs. To probe the particle motion directly, a 3D particle tracking experiment was conducted using field-dependent confocal microscopy. To enable the particle tracking, the surface of the carbonyl iron particles was modified prior to synthesizing an otherwise identical version of the ultrasoft MRE sample 1. The modification process consisted of three major stages (**Fig. S5**). First, carbonyl iron powder (CIP) was covered with a silica layer prepared via tetra-ethoxysilane (TEOS) hydrolysis. Next, the particles (CIP/SiO_2_, CIP-TEOS) were coated with a second layer formed through hydrolysis and condensation of (3-Aminopropyl)-triethoxysilane (APTES). Finally, a fluorophore was attached to the CIP-TEOS-APTES particles through a bioconjugation of Atto 488 NHS ester.

In the first step of the magnetic particle modification, a silica precursor consisting of anhydrous ethanol, TEOS, and hydrochloric acid were mixed in a molar ratio of 7.6:1:0.05, where hydrochloric acid was added as a catalyst^4^. The mixture was stirred for 2 h at room temperature. The resulting TEOS hydrolysate was kept for 24 h for sol aging at room temperature. We then combined 20 g of CIP with 50 mL anhydrous ethanol and homogenized the mixture in a water bath using ultrasonic vibration for 10 min. Then, the pre-prepared TEOS hydrolysate was dropwise added using a dropping funnel into CIP mixture with mechanical stirring at room temperature for 3 h. Additionally, the dispersion was homogenized mid-reaction and post-reaction for 10 min, respectively. The resulting particles were collected using a permanent magnet and washed 3 times with anhydrous ethanol, to remove organic residues and prevent the particles from agglomerating. The product (CIP-TEOS) was dried at 60 °C for 24 h and collected.


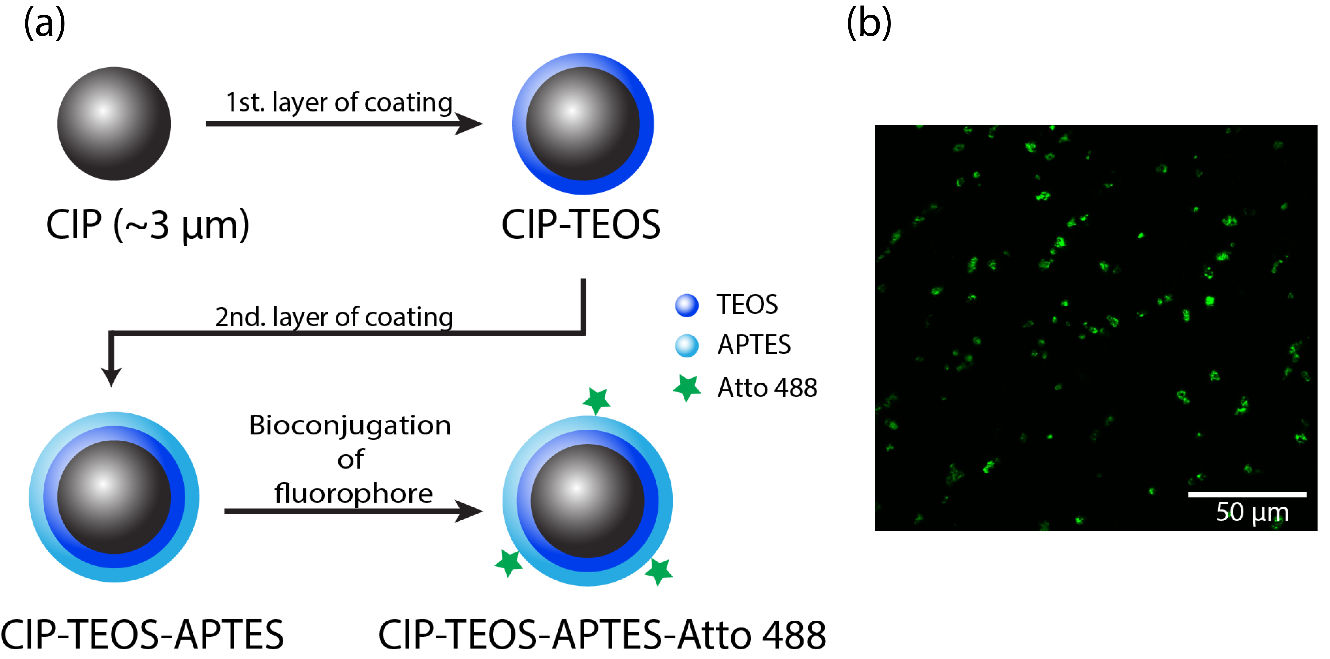


**Fig. S5** (a) Schematic of fluorescent labelling process of iron particles. (b) Fluorescent image of fluorescently labeled iron particles.

To cover the CIP-TEOS with APTES, we first equipped a 100 mL, three-neck, round bottom flask with an overhead mechanical stir through the middle neck, cold water condenser and thermometer through the outer necks. 10 g of prepared CIP-TEOS was combined with 80 mL dry toluene and pre-homogenized using ultrasonic vibration in water bath for 30 min. The mixture was poured in the three-neck, round bottom flask followed by injecting a solution of 1.2 mL of APTES in 5 mL dry toluene via syringe under vigorous mechanical stirring. The reaction mixture was heated to 105 °C for 6 h. Aluminum foil was used to cover the flask and heat block to maintain the insulation during the reaction. The powder was collected by using a permanent magnet upon cooling to room temperature. The APTES coated CIP-TEOS (CIP-TEOS-APTES) was washed three times with anhydrous ethanol. The product (CIP-TEOS-APTES) was dried at 60 °C for 24 h and collected.

Finally, we used the primary amine reactive Atto 488 NHS ester to label CIP-TEOS-APTES through conjugation with the amine group on APTES. The stock solution of Atto 488 NHS ester was prepared by dissolving 1 mg of Atto 488 NHS ester in 100 µL of anhydrous DMSO. To bind fluorophores on the surface of CIP, 500 mg of CIP-TEOS-APTES were dispersed in 9.99 mL of anhydrous DMSO followed by adding 10 µL of stock solution of Atto 488 NHS ester. To prevent photobleaching, the reaction was completed in the dark for 6 h at room temperature. The Atto 488 NHS ester labeled CIP-TEOS-APTES was magnetically separated and washed five times with anhydrous ethanol to remove non-conjugated fluorophores. The final product (CIP-TEOS-APTES-Atto 488) was dried at 60 °C for 24 h in the dark and collected.

**Fig. S6** shows the experimental setup used for the 3D particle tracking confocal microscopy measurements. The volume constrained fluorescently labeled MRE sample was placed above the confocal microscope objective. An electromagnet (from GMW) was connected to an Agilent 6553A power supply operating in constant current mode. The current was tuned to apply fixed magnetic fields of 0, 250 and 500 Oe to the MRE sample, measured using a gaussmeter (Lakeshore®-410). Z-stack images were taken at each magnetic field using a Zeiss LSM880 confocal microscope with a 20x/1.0 water immersion objective lens. The resolution of each z-stack was 1772x1772x28 pixels^3^ with a voxel size of 200x200x410nm^3^ and the acquisition time for a full stack was thirty seconds.

**Fig. S6** Experimental setup for the magnetic-field dependent confocal microscopy of an MRE containing fluorescently labeled iron particles.


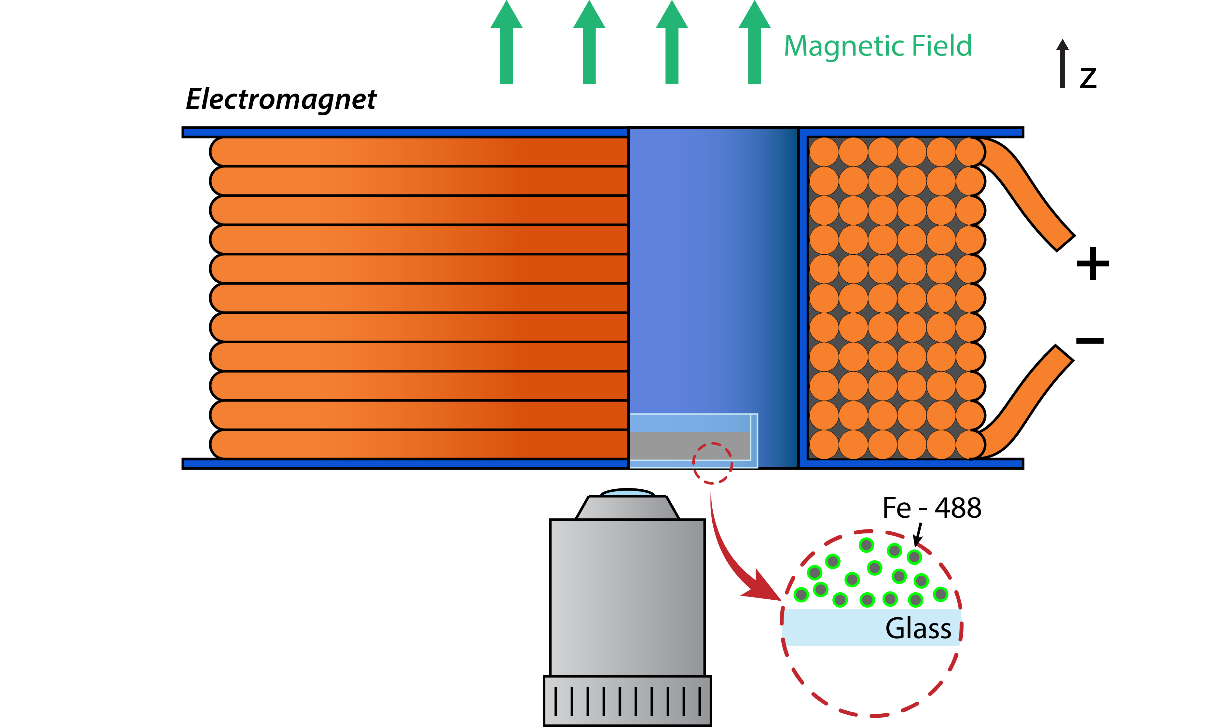


The trajectories of six particles were tracked as the magnetic field was increased from 0 to 500 Oe and back to 0 Oe in steps of 250 Oe. First, an in-focus image was obtained prior to applying any magnetic field, and images cropped around each particle were selected. A set of 2D cross correlation were performed using the cropped in-focus images of each of the selected particles as inputs to determine the in-focus 3D position of each particle at every magnetic field. The magnetic field-dependent motion for one of the particles is shown in **Fig. S7**. The particle moves primarily along the applied magnetic field direction, and the magnitude of the particle motion is larger when the $H$ is increased from 250 to 500 Oe than it is for the 0 to 250 Oe field step, and the motion is several microns in magnitude, all of which are in qualitative agreement with the modeling results. The microscopy results hence confirm by direct observation that the iron particles exhibit micron-scale motion primarily along the applied magnetic field direction within the polymer. In addition, **Fig. S7** indicates that the field-induced particle motion is reversible within the measurement uncertainty.


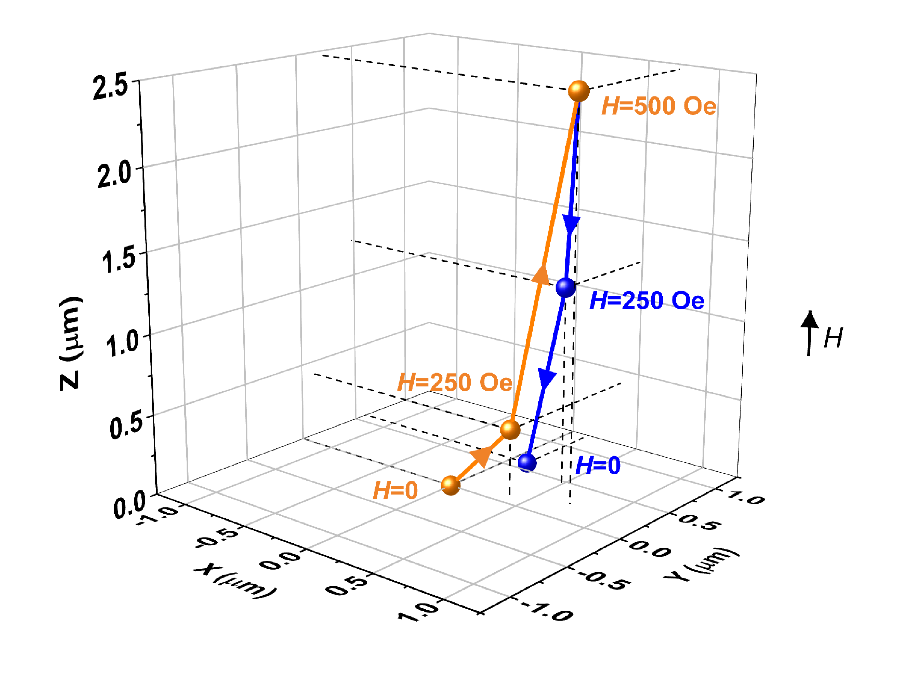


**Fig. S7** Trajectory of an iron particle within the polymer matrix of an ultrasoft MRE extracted using confocal microscopy. The measurements were done on the fluorescently labeled MRE sample 1, and $H$ was applied along the Z direction. The uncertainty for the z-position is $\pm0.4$ $\mu$m and that for the x- or y-position is $\pm1 \mu$m.

1. **Modeling for field applied at an intermediate angle**

The model of two dipoles connected by a single spring, which was used to obtain the results shown in **Figs. 4** and **5,** is suitable for studying the response of magnetic dipoles to a magnetic field that is applied along or perpendicular to the line that connects the centers of the two particles. We refer to the above case as the on-axis case. To capture the restoring force in response to a rotational motion, we employed a three-spring model, as demonstrated by Puljiz et al^5^ and illustrated in the inset of **Fig. S8**, where the gray regions that the two end springs attached to are the equilibrium positions of the spheres at zero magnetic field. **Fig. S8** shows modeling results done using this three-spring approach with the field applied at an angle of $\theta=19^{\circ}$ to the particle axis with $k=4\times{10}^{-3}$ N/m, $S_{0}=9 \mu m$, and otherwise the same parameters as used in **Figs.** **4** and **5**. We note that Puljiz et al.^5^ describe a method to modify the $k$ values for the two additional end springs to capture the shear modulus magnitude more accurately. Here we use the same$k$ to provides a reasonable qualitative picture of the response. As shown in **Fig. S8,** at this angle ($\theta=19^{\circ}$), the net force is attractive and the particles consequently move towards each other at intermediate fields and also rotate towards the applied field direction. As in the on-axis case, the hysteretic region shown in **Fig. S8b** corresponds to the region of bistability.

**SM References**


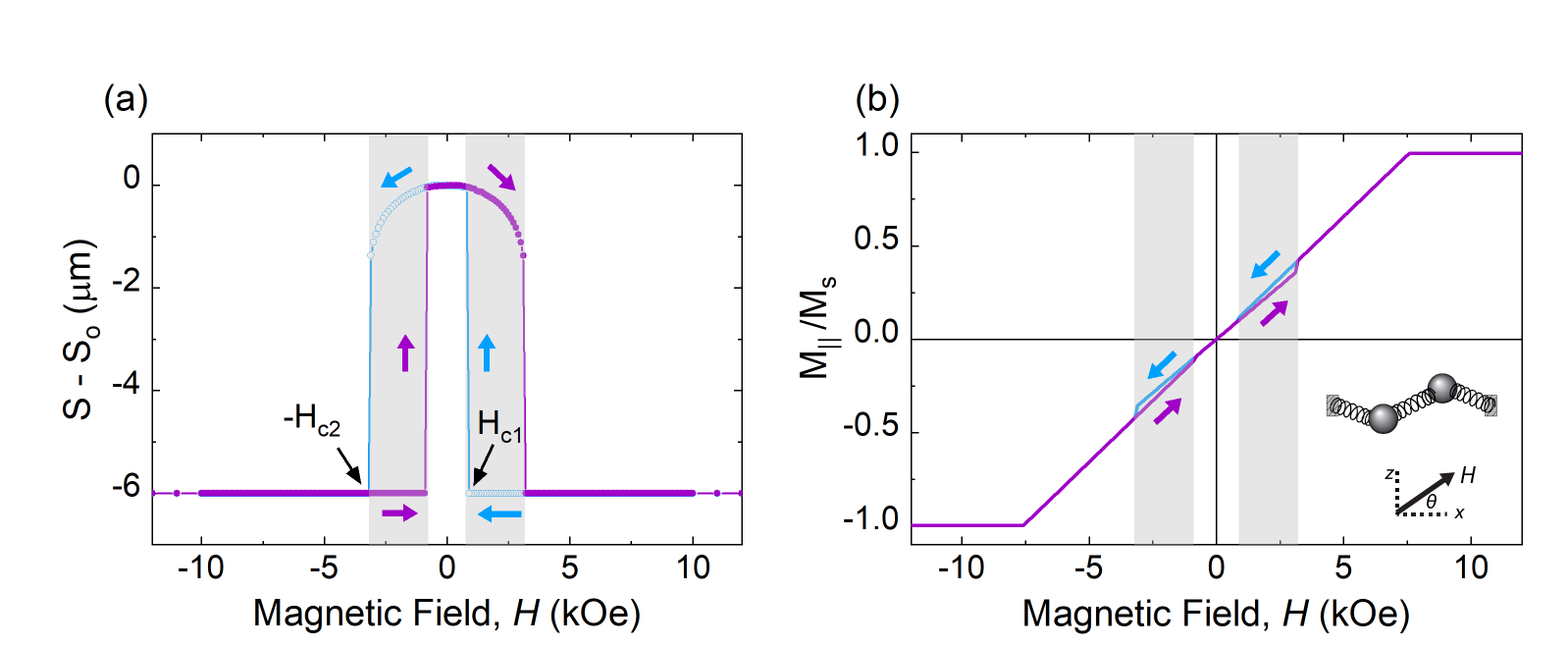


**Fig. S8** Modeling results for a field angle of $\theta= 19^{\circ}$, $k = 4 \times{10}^{-3}$ N/m for the three springs, $S_{0}= 9 \mu m$ for the spring that connects the two dipoles, $R = 1.5 \mu m$ ($\chi= 2$, $M_{s}=1.4 \times{10}^{6}$ A/m). As shown in the inset, one spring connects the particles and two additional springs connect each particle to its equilibrium position. The magnetic response parallel to the applied field and the particle displacement magnitude ($S - S_{0})$ are shown in (a) and (b), respectively. The field ranges over which particle bistability occurs are shaded in (a) and (b), and in (b) the bistability field range (shaded in gray) corresponds to the field range over which magnetic hysteresis is observed.

^1^ A.C. Rennie, P.L. Dickrell, and W.G. Sawyer, Tribology Letters **18**, 499 (2005).

^2^ K.D. Schulze, A.I. Bennett, S. Marshall, K.G. Rowe, and A.C. Dunn, Journal of Tribology **138**, (2016).

^3^ K.L. Johnson, Proc. Inst. Mech. Eng. **223**, 254 (2009).

^4^ P. Małecki, K. Kolman, J. Pigłowski, J. Kaleta, and J. Krzak, Journal of Solid State Chemistry **226**, 224 (2015).

^5^ M. Puljiz, S. Huang, K.A. Kalina, J. Nowak, S. Odenbach, M. Kästner, G.K. Auernhammer, and A.M. Menzel, Soft Matter **14**, 6809 (2018).
